# The Evolution of Cardiac Rehabilitation from Supervised Models to New Frontiers in Digital Health

**DOI:** 10.3390/jcm15072515

**Published:** 2026-03-25

**Authors:** Alfredo Mauriello, Adriana Correra, Anna Chiara Maratea, Vincenzo Russo, Biagio Liccardo, Felice Gragnano, Vincenzo Acerbo, Arturo Cesaro, Mario Pacileo, Carmine Riccio, Paolo Calabrò, Antonello D’Andrea

**Affiliations:** 1 S.C. Cardiology, Institute National Cancer, IRCCS, Foundation “G. Pascale”, Via M. Semmola 52, 80131 Naples, Italy; alfredo.mauriello93@libero.it; 2 Cardiology Department, Ospedali Riuniti University Hospital, Viale Pinto 1, 71122 Foggia, Italy; adrianacorrera@gmail.com; 3 Department of Cardiovascular Disease, ASL Napoli 1 Centro, Via Comunale del Principe, 13/a, 80145 Napoli, Italy; annachiara.maratea@gmail.com; 4 Cardiology Unit, Department of Medical and Translational Sciences, University of Campania “Luigi Vanvitelli”, “V. Monaldi” Hospital, Via Leonardo Bianchi snc, 80131 Naples, Italy; vincenzo.russo@unicampania.it (V.R.); liccardob@gmail.com (B.L.); 5 Division of Cardiology, Department of Medical and Translational Sciences, University of Campania “Luigi Vanvitelli”, Azienda Ospedaliera Sant’Anna e San Sebastiano, Via Palasciano Ferdinando 1, 81100 Caserta, Italy; felice.gragnano@unicampania.it (F.G.); arturo.cesaro@unicampania.it (A.C.); carminericcio8@gmail.com (C.R.); paolo.calabro@unicampania.it (P.C.); 6 Cardiology and Intensive Care Unit, Department of Cardiology, “Umberto I” Hospital, Via Alfonso De Nicola 1, 84014 Nocera Inferiore, Italy; mario.pacileo@aslsalerno.it

**Keywords:** cardiac rehabilitation, telerehabilitation, artificial intelligence, cardiac disease, heart failure, acute coronary syndrome

## Abstract

**Background/Objectives**: Cardiac rehabilitation (CR) is a cornerstone of secondary prevention, traditionally delivered through supervised center-based models. However, significant logistical barriers and high healthcare costs necessitate a paradigm shift. This review aims to assess the impact of emerging digital frontiers, specifically telerehabilitation (CTR) and artificial intelligence (AI), on overcoming these challenges and improving clinical outcomes. **Methods**: This study is a narrative, clinically oriented review informed by a structured search of PubMed/MEDLINE and EMBASE for literature published between January 2015 and January 2026. **Results**: Evidence indicates that CTR is non-inferior to center-based programs in terms of exercise capacity and quality of life (QoL). Digital tools, such as wearable devices and mobile health (mHealth) applications, have significantly increased program participation and improved adherence to lifestyle modifications. Furthermore, the integration of AI facilitates early detection of cardiac events and personalized exercise prescription, while prehabilitation models have been shown to reduce postoperative hospital stays. **Conclusions**: Digitalization of CR may represent a cost-effective alternative that bridges the gap in global access. While technology serves as an essential diagnostic partner, a robust regulatory and privacy framework is required to protect data sovereignty. Ultimately, multidisciplinary synergy between human expertise and digital innovation is important for providing an equitable and personalized pathway to recovery.

## 1. Introduction

**Figure 1:**
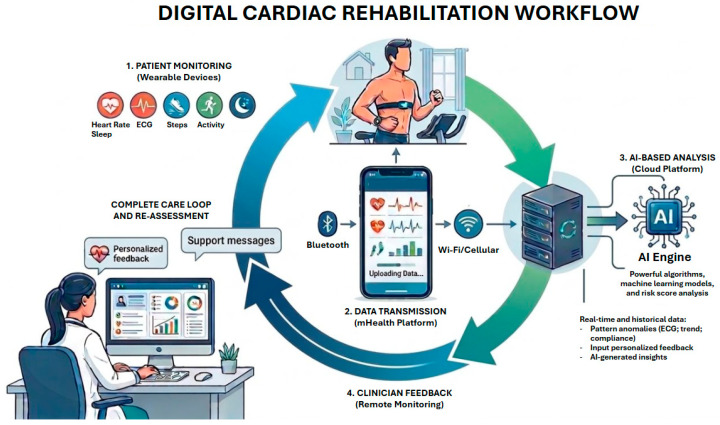
Digital cardiac rehabilitation workflow. AI: artificial intelligence; ECG: electrocardiogram.

Cardiac rehabilitation (CR) is defined as exercise practices for patients in secondary prevention after coronary artery bypass graft (CABG) surgery, myocardial infarction (MI), stable angina, percutaneous coronary intervention (PCI), symptomatic peripheral arterial disease (PAD), cardiac transplantation, chronic heart failure with reduced ejection fraction (HFrEF), heart valve surgery, and cardiotoxicity, to reduce cardiovascular mortality, reduce risk of hospital admission, and improve exercise capacity and health-related quality of life (QoL) [1,2]. However, in recent years, the CR paradigm has been shifting. Today, CR is defined as a multidisciplinary program that includes exercise training, cardiac risk factor modification, psychosocial assessment, and outcome assessment [3]. The increased utilization of CR has led to higher costs for the public health system. However, the implementation of systems focused on home-based programs has enabled cost reductions and improved accessibility to CR [4]. This comprehensive review aims to assess the impact of emerging CR paradigms powered by telemedicine and artificial intelligence (AI). Figure 1 represents an illustration of the digital cardiac rehabilitation workflow.

## 2. Materials and Methods

This article is conceived as a narrative, clinically oriented review exploring the contemporary landscape of CR and its evolution from traditional supervised models toward digital frontiers. Rather than producing a formal meta-analysis, the primary aim of this work is to provide a pragmatic synthesis of emerging evidence regarding telerehabilitation (CTR) and AI, highlighting how these tools address current logistical barriers and improve patient outcomes.

To ensure transparency, a structured search strategy was employed to inform the narrative synthesis process. A comprehensive search was conducted in PubMed/MEDLINE and EMBASE for articles published between January 2015 and January 2026. The search utilized combinations of keywords including “cardiac rehabilitation,” “telerehabilitation,” “artificial intelligence,” “mHealth,” and “digital health”. Document selection prioritized English-language articles, including systematic and narrative reviews, retrospective and prospective studies, and randomized controlled trials (RCTs) that addressed the efficacy, cost-effectiveness, and accessibility of digital CR paradigms. While this process does not strictly adhere to the rigorous replicability of a formal systematic review, this methodology ensures that the narrative synthesis is evidence-based and encompasses the major clinical and scientific developments related to the multidisciplinary heart rhythm team.

## 3. Traditional Model and Its Limits

The current CR model consists of four phases, including the presence of patients in the health structure.

### 3.1. Phase I: The Inpatient Phase (Acute)

Phase I of cardiac rehabilitation commences within the inpatient setting during the immediate post-acute stage of the disease. The primary clinical objectives focus on hemodynamic stabilization and the implementation of early mobilization protocols. During this stage, the multidisciplinary team prioritizes targeted educational interventions to enhance the patient’s understanding of the pathology, alongside assessing exercise tolerance during the functional transition from bed rest to ambulation. Furthermore, the hospitalization period represents a critical window for psychological intervention, essential for facilitating the processing of diagnostic trauma and ensuring a secure, informed transition toward discharge and subsequent rehabilitative phases [5].

### 3.2. Phase II: The Subacute Phase (Outpatient)

Upon hospital discharge, patients transition into the subacute phase of CR, which is characterized by a high-intensity, multi-component intervention. This phase typically encompasses a duration of 12 to 24 weeks within a supervised outpatient facility. The primary clinical framework consists of electrocardiographically (ECG)-monitored aerobic and resistance training tailored to the patient’s functional capacity. A cornerstone of this phase is comprehensive risk factor stratification and modification. This multidisciplinary approach integrates nutritional therapy, tobacco cessation protocols and psychosocial intervention. This integrative model aims to optimize cardiovascular efficiency while concurrently arresting the progression of atherosclerotic disease [6].

### 3.3. Phase III: The Intensive Outpatient Phase (Transition)

Upon completion of the supervised outpatient program, patients progress to Phase III, a clinical bridge to long-term independence. During this period, the frequency of direct clinical supervision and continuous telemetry is systematically titrated downward. The therapeutic focus pivots toward the development of self-regulatory competencies. The overarching objective of Phase III is the consolidation of lifestyle modifications, ensuring that cardioprotective behaviors are transitioned from monitored intervention to a permanent, self-sustained habits [7].

### 3.4. Phase IV: The Maintenance Phase (Life-Long)

This phase is the patient’s commitment to a permanent lifestyle of secondary prevention. Phase IV represents the maintenance stage of CR, characterized by its indefinite duration and the absence of direct clinical supervision. The transition from Phase III to Phase IV marks the shift from supervised intervention to patient-led secondary prevention. Phase IV is the practical application of chronic disease management, where the individual utilizes self-monitoring strategies to ensure cardiovascular stability and enhance overall QoL [8].

This model has several limitations, particularly logistical ones. Specifically, the number of patients requiring treatment is excessively high for most healthcare organizations. Furthermore, patients must physically travel to the facility to undergo the CR program; this increases time and costs, reducing adherence, diminishing its benefits, and increasing overall risks. However, new models of CR have been proposed [4].

Shields et al. [4], in their systematic review including nine studies, showed that center-based CR is recognized as cost-effective for individuals following a cardiac event. However, home-based alternatives were less expensive for patients.

Sugiharto et al. [9], in their systematic review including 23 studies, showed that barriers that contribute to low participation are classified into five categories. These barriers include individual, health history, environmental, logistical, and health system factors. The most common barriers in each category were age, comorbidities, lack of social support, distance or travel time, and cost and economic status.

Braver et al. [10], in their cost-effectiveness analysis, showed that digitally enabled cardiac rehabilitation is a cost-effective alternative to usual care, offering improved health outcomes at an acceptable cost.

## 4. Technological Innovation: Digital Health

**Figure 2:**
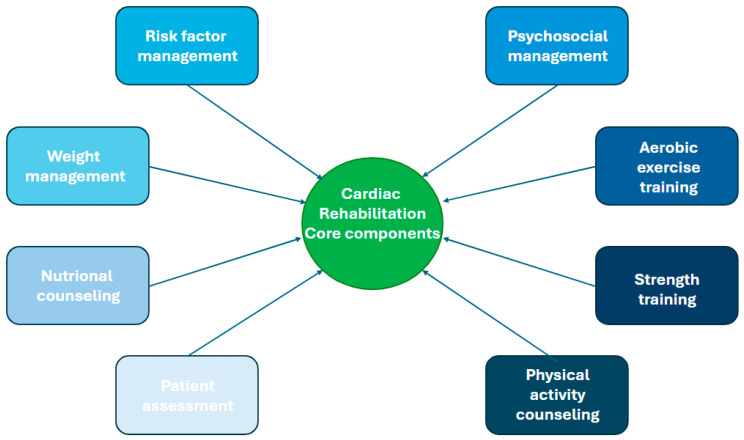
Cardiac rehabilitation core components.

Cardiac telerehabilitation (CTR) is defined as the delivery of RC services using digital health technologies and telecommunications to support patients at home. It encompasses a range of interventions, including all CR core components, provided through video conferencing, remote monitoring, mobile applications, and other digital platforms to enhance access, convenience, and continuity of care. Figure 2 represents CR core components.

### Two Models of CTR Exist

Fully remote CTR: center-based CR is completely replaced by CTR. There are no center-based CR sessions for patients.

Hybrid CTR: In a full-content hybrid CTR model, all core components are either delivered remotely or at the CR center.

Within the world of remote recovery, two distinct paths emerge—the synchronous and the asynchronous—each offering a different support.

Synchronous CTR is defined as a real-time interaction, where a physiotherapist might guide a patient through an exercise session via a video link. To ensure safety from a distance, the patient’s home becomes a mini clinic, where specialized tools are installed, such as tele-ECG monitors, blood pressure cuffs, and digital scales, all streaming vital data instantly through a smartphone [11].

On the other hand, asynchronous CTR operates at a different time, focusing on the power of self-management. Patients document their physiological metrics and transmit them to the clinical facility for periodic professional review, with physician intervention triggered specifically by automated threshold alerts [12]. This approach offers several compelling advantages, such as autonomy, use of scales and accessibility [13].

The integration of synchronous and asynchronous elements through a mixed-method approach enhances the versatility of the CR experience. This paradigm utilizes live interactive sessions for specialized consultations, complemented by the periodic submission of patient-generated data for continuous monitoring. This dual-modality structure not only reinforces the patient–clinician relationship but also facilitates a persistent, asynchronous tracking of lifestyle modifications.

In recent years, home-based CR, delivered by CTR, has been suggested as an alternative or adjunct to center-based RT to increase access, adherence, and participation rates [14]. By integrating tools like tele-coaching, social networking, remote monitoring, and e-learning, CTR serves as a comprehensive digital bridge that covers nearly every fundamental pillar of traditional CR [15]. Beyond simply boosting initial participation following a heart-related event, CTR acts as a vital bridge to long-term health. It breaks the mold of traditional CR, which is often confined to a brief window of 24 to 36 sessions [16].

The Home-versus center-based EXercise InTervention in patients with Heart Failure (EXIT-HF) [17] was a non-inferiority randomized clinical trial (RCT) including 120 patients (age 62 ± 11 years, 66% men, mean left ventricular ejection fraction 36 ± 11%) comparing CTR for patients with HFrEF and heart failure with preserved ejection fraction (HFpEF) with center-based CR. The study demonstrated the non-inferiority of CTR in terms of exercise capacity (0.8 mL/kg/min [95% CI: 1.8 to −0.16 mL/kg/min]; *p* = 0.10) and QoL (0.15 points [95% CI: −6.6 to 6.9]; *p* = 0.97).

At the same time, Tegegne et al. [18], in their meta-analysis including 132 RCTs (*n* = 18,670), showed that CTR was associated with improvements in exercise capacity; however, no improvement in HF-related hospitalization or mortality risk was observed (odds ratio (OR) = 0.41 (0.17 to 0.76) and OR = 0.42 (0.16 to 0.90), respectively).

Regarding other indications of CR, the results are similar. McDonagh et al. [19], in their systematic review including 24 trials with a total of 3046 participants undergoing CR with MI, angina, HFrEF, or revascularization, showed that no evidence of a difference was seen between home- and center-based CR in their primary outcomes, including cardiac mortality, cardiac events and QoL, up to 12 months of follow-up: total mortality (risk ratio [RR] = 1.19, 95% confidence interval [CI] 0.65 to 2.16) or exercise capacity (standardized mean difference (SMD) = −0.10, 95% CI −0.24 to 0.04). The majority of evidence showed no significant difference in health-related QoL up to 24 months follow-up between home- and center-based CR. Table 1 summarizes main trials regarding CRT.

## 5. Devices for Cardiac Telerehabilitation

There are two main types of remote devices used for remote CR: ergometers and wearable watches

The implementation of remote CR via home-based ergometry necessitates the installation of specialized equipment and internet-connected interfaces, enabling real-time physiological surveillance by clinical staff during physical exertion. While this modality ensures a high degree of safety through continuous professional monitoring of the patient’s hemodynamic status, several logistical and economic challenges persist. Specifically, synchronous monitoring imposes a significant administrative and temporal burden on healthcare personnel and requires adequate domestic space for equipment housing. Furthermore, the requisite technical proficiency and the substantial costs associated with these proprietary devices may limit their accessibility and widespread clinical adoption, particularly among patients with lower digital literacy or limited financial resources [20].

Wearable devices are superior for data collection and analysis; furthermore, they are versatile, with extensibility for functional improvements and new features for apps [21]. The Apple Watch (Apple Inc.^®^, Cupertino, CA, USA) is a widely used wearable watch globally, and several other wearable devices have recently been commercialized.

A South Korean report suggested that a home CR program using a portable ECG data transmitter (HeartCallTM^®^) improved participants’ exercise tolerance and QoL [22].

**Figure 3:**
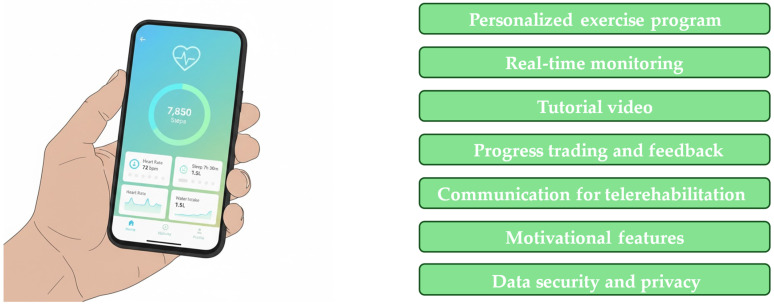
Software as a medical device for remote cardiac rehabilitation.

Remote CR frameworks leverage dedicated mHealth platforms to enable patients with cardiovascular disease to execute exercise protocols under remote clinical supervision. These digital ecosystems typically integrate individualized prescription algorithms, real-time physiological tracking, and telehealth modalities to ensure both safety and adherence [23]. Data is transmitted to the platform in real time during training sessions, allowing healthcare providers to remotely monitor the patient’s progress and make necessary adjustments [23]. Table 2 summarizes candidate patients for remote CR. Figure 3 represents the advantages of using the software as a medical device.

## 6. Use of Apps and Artificial Intelligence

Mobile health technology (mHealth) can foster the development of technologies that can provide health advice, such as step counting, calorie consumption, and dietary recommendations. In addition, use of mHealth with artificial intelligence can be used for detecting atrial fibrillation [24], prescribing physical exercise [25] and risk prediction and early decompensation detection in heart failure [26]. This approach could be attractive because it is easy to use, thus reducing healthcare costs [27]. Cruz-Cobo et al. [28], in their RCT including 287 patients (69.0% males, mean age of 62.53 years), showed that significant improvements were observed in the mHealth group (eMotiva^®^) compared with the control group at 6 months in terms of adherence to the Mediterranean diet (<0.001) and frequency of eating foods (all *p* < 0.001); (2) physical activity (*p* < 0.001) and sedentary time (*p* < 0.001); (3) exercise capacity (*p* = 0.04); and (4) level of knowledge (*p* < 0.001).

At the same time, Rivers et al. [29], in their prospective study including 204 patients, showed that CR participation improved from 21% (95% CI 14–30%) to 63% (95% CI 53–71%) with the addition of the app (*p* < 0.001) (Cardihab^®^).

In addition to mHealth, some applications integrate an AI system, improving outcomes and allowing for personalized CR programs.

Sotirakos et al. [30] conducted a systematic review including eight studies that showed that the incorporation of AI into CR delivery led to early detection of cardiac events, allowing for home-based monitoring and improved clinician decision-making.

Saklica et al. [31], in their RCT including 52 patients with coronary artery disease, showed that patients assigned to the group that used a program of CR detected by AI, after a 12-week program, had significant exercise capacity improvements compared to the control group (*p* = 0.001), an increase in adherence (*p* < 0.001), and an increase in patient-reported satisfaction (*p* < 0.001).

The Rehabilitation through Exercise prescription for Cardiac patients using an Artificial Intelligence web-based Programme (RECAP) [32] is an ongoing RCT that includes 70 patients undergoing a home-based CR program delivered using a mobile app with AI to prescribe exercise goals to the participants every week, with primary outcomes that include recruitment rates, dropout rates, user satisfaction, and 6 min walking test (6MWT).

## 7. Novel Clinical Paradigm

### 7.1. Prehabilitation

In recent years, the concept of prehabilitation, the adoption of behavioral interventions before surgery to enhance postoperative outcomes, has gained significant momentum. While this approach was initially pioneered and studied extensively in oncology patients, its application is now expanding into other medical fields, including CR [33].

Wang et al. [34], in their systematic review and meta-analysis including 31 studies enrolling a total of 2895 participants who underwent cardiac surgery, showed that preoperative exercise training intervention is significantly associated with a lower risk (OR: 0.15, 95%CI: 0.06, 0.38) of composite postoperative pulmonary complications. Therefore, preoperative exercise training intervention reduced the postoperative hospital stay by −1.57 days (95% CI: −2.33, −0.81). Finally, preoperative exercise training significantly decreased the postoperative intensive care unit stay by −2.22 h (95% CI: −3.05, −1.38) and hospital stay by −1.82 days (95% CI: −3.38, −0.27).

Hurtado-Borrego et al. [35], in their systematic review including nine studies enrolling a total of 873 patients who underwent cardiac surgery, showed that prehabilitation significantly improved functional capacity (∆6MWT: +52.4 m; *p* < 0.001), reduced respiratory complications and accelerated hospital discharge (−15.2 h; *p* < 0.001).

Finally, Yau et al. [36], in their single-center RCT including 138 (median age 64 with range 60–69 years; 70% males), showed that prehabilitated patients had lower disability levels than control patients (*p* = 0.022) at 90 days after surgery.

### 7.2. Multidisciplinary Approach: Nutrition, Stress Management and Smoking in the Digital Era

Over the last two decades, cardiovascular health guidelines have undergone a critical transition from nutrient-centric targets to recommendations for integrated dietary patterns [37]. This shift reflects a deeper understanding of the synergistic effects of bioactive food components. Longitudinal studies have confirmed that the overall quality of dietary patterns is inversely correlated with the risk of recurrent complications in high-risk populations, consolidating pattern-based dietetics as a cornerstone of secondary prevention [38].

In recent years, most CR programs have included nutrition interventions, and 80% have included a dietitian as part of the program staff [39].

However, several mHealth applications are developing CR programs, including nutrition care. These apps are developed to improve lifestyle changes in healthy patients [40], but they could be applied in the future in CR. We have discordant mHealth data on the use of apps in CR.

Gallagher et al. [41] conducted a single-blind RCT evaluating the effect of a game-based mobile app intervention (MyHeartMate^®^) on cardiovascular risk factors and lifestyle behaviors, including nutrition. The primary outcome was self-reported physical activity, and the secondary outcomes included lipid levels, blood pressure, body mass index, and smoking. Over 6 months, no differences were reported for the primary outcome (95% CI: −37.4, 696; *p* = 0.064). No differences were observed between the groups for the secondary outcomes, except for lower triglyceride levels in the intervention group (*p* = 0.004). Therefore, future research should focus on this field.

Stress management is currently excluded from routine CR protocols due to a lack of clinical consensus and methodological inconsistencies in the literature exploring the causal link between psychological stress and coronary artery disease. Nevertheless, emerging evidence suggests that integrating psychosocial interventions into CR can yield significant improvements in long-term cardiovascular outcomes. Shi et al. [42], in their systematic review including nine studies, suggested the benefits of stress management in patients undergoing CR. Therefore, based on RCTs, stress management could be considered an active component of CR.

Also, for this component of CR, a digital system has been evaluated. Cortes-Perez et al. [43], in their systematic review including eight RCTs enrolling 510 patients in total, showed that immersive virtual reality, especially when combined with a traditional CR, reduces depression (*p* < 0.001), anxiety (*p* = 0.006), and stress (*p* < 0.001) in patients with cardiovascular disease.

## 8. Special Population

Within numerous high-income jurisdictions, including the United States and the United Kingdom, significant disparities in CR uptake persist. Participation rates remain disproportionately low among specific demographic cohorts, most notably the elderly and individuals from lower socioeconomic backgrounds [44,45,46,47].

Also, for frail and elderly patients, CR improves cardiovascular outcomes. Baldessaroni et al. [48], in their observational study including 160 patients (mean age 80 ± 4 years) after an acute coronary event or cardiac surgery, showed that an exercise-based CR program was associated with improvements in all domains of physical performance (VO2 peak: OR = 0.86, 95% CI = 0.77–0.97; 6MWT: OR = 0.99, 95% CI = 0.99–1.00; peak torque: OR = 0.96, 95% CI = 0.94–0.98).

Therefore, in this patient population, the home-based CR program appears to offer greater benefits than the center-based program. Snoek et al. [49], in their RCT including 179 patients with median age of 72 (range, 65–87) years, showed that a 6-month home-based CR program for patients 65 years or older with coronary artery disease or a valvular intervention was safe and beneficial in improving VO2 peak when compared with no CR (+0.9 [95% CI, 0.05 to 1.8] mL/kg^−1^/min^−1^).

Frailty, defined as the incapacity to adapt to external stressors, among CR participants, is associated with worse health outcomes. However, even if the prevalence of frailty is high in CR and is associated with a greater mortality risk, CR improves frailty and physical health outcomes. MacEachern et al. [50], in their systematic review including 34 studies enrolling 19,360 patients with a high prevalence of frailty (46%), showed that frailty improved following CR participation (SMD: 0.68, 95% CI 0.37–0.99; *p* < 0.0001).

Also, home-based CR improves outcomes for these patients. Nagatomi et al. [21], in their single-center, open-label RCT including 30 outpatients with chronic HFrEF (mean age 63.7 ± 10.1 years, 53% male), showed that the use of CTR in patients with physical frailty improved exercise tolerance and improved lower-extremity muscle strength (*p* < 0.001)

## 9. Future Perspectives

In terms of clinical equivalence, CTR appears non-inferior to center-based CR, particularly in low-to-moderate-risk stable patients, with robust improvements in functional endpoints such as VO2 peak and QoL. However, evidence remains weaker for long-term mortality and heart-failure-related hospitalizations. A challenge remains in the digital divide; while CTR aims to broaden access, the requirement for expensive hardware and high digital literacy may inadvertently exclude elderly or socioeconomically disadvantaged populations. Furthermore, safety concerns regarding immediate emergency response for high-risk patients and the current lack of a harmonized regulatory framework for AI-driven data management persist as significant barriers to large-scale implementation. As we look toward the horizon of cardiovascular care, the evolution of CR is increasingly defined by sophisticated synergy between human expertise and digital innovation. While the emergence of AI-driven CTR and wearable sensors offers unparalleled precision in real-time physiological monitoring, often surpassing traditional supervised models in data granularity and longitudinal adherence, the human clinician remains an irreplaceable anchor for complex clinical reasoning and the therapeutic relationship. Future paradigms will likely shift from technology to an essential diagnostic partner, yet this transition necessitates a robust regulatory framework to address the gray areas of algorithmic accountability and data sovereignty. From a privacy perspective, implementing General Data Protection Regulation (GDPR)-compliant systems and advanced encryption is critical to mitigate the risks inherent in large-scale health data harvesting. Economically, the digitalization of CR is forecasted to be a transformative catalyst; by reducing hospital readmissions and optimizing resource allocation, virtual and hybrid models offer a cost-effective alternative that could bridge the gap in global access to secondary prevention. However, to reduce the cost of digital CR, a hybrid model or less expensive mHealth apps could be used. Ultimately, the future of the field lies in ensuring that these digital advancements provide an equitable, high-quality, and personalized pathway to recovery.

## 10. Conclusions

The digitalization of CR may represent a transformative catalyst for modern cardiovascular care, offering a cost-effective alternative capable of bridging the global gap in access to secondary prevention. The scientific evidence analyzed may show that CTR models are non-inferior to traditional programs. Therefore, while technological innovation, supported by AI and wearable devices, is now established as an essential diagnostic partner for precision physiological monitoring, the clinician remains an irreplaceable anchor. However, the full integration of these models requires robust regulatory frameworks that ensure data sovereignty and compliance with GDPR requirements. Furthermore, the successful large-scale implementation of these digital systems requires dedicated healthcare funding and specialized training programs for medical professionals to ensure clinical proficiency and technological literacy. In conclusion, the future of this field lies in multidisciplinary synergy between human expertise and digital innovation.

## Figures and Tables

**Table 1:** Main features of studies regarding CRT.

Trial	Delivery	Population or Studies (N)	Outcome	Results
EXIT-HF	RCT	120	Change in peak oxygen uptake (VO2peak) and QoL	Demonstrated non-inferiority of CTR in terms of exercise capacity and QoL
McDonagh et al. [19]	Meta-analysis	24 trials	Cardiac mortality, cardiac events and quality of life	No evidence of a difference
Tegegne et al. [18]	Meta-analysis	132 RCTs	Exercise capacity and HRQoL	CTR was associated with improvements in exercise capacity; however, no improvement in HF-related hospitalization or mortality risk was observed

CTR: cardiac telerehabilitation; EXIT-HF: Home-versus center-based EXercise InTervention in patients with Heart Failure; HRQoL: health-related quality of life; OR: odds ratio; RCT: randomized controlled trial; SMD: standardized mean difference.

**Table 2:** Patients with an indication for remote cardiac rehabilitation.

Patient Category	Clinical Indication	Critical Rationale and Bias Analysis
Low-to-Moderate Risk	Stable patients post-PCI or with compensated HFrEF	Robust Equivalence: In these subjects, CTR is non-inferior to center-based CR regarding functional capacity and quality of life.
Geographically Remote	Patients in rural areas or residing far from specialized centers	Overcoming Barriers: CTR reduces travel costs and logistical burdens, significantly improving global accessibility and long-term participation.
Digitally Literate	Patients proficient in using smartphones and mHealth applications	Risk of Selection Bias: There is a high risk of excluding the geriatric or less-educated population (digital divide), potentially limiting the generalizability of the findings.
No Frailty or Sarcopenia	Patients with preserved baseline motor autonomy	Functional Endpoints: CTR is highly effective in enhancing muscle strength and physical performance in non-frail individuals.
Busy Patients	Working-age individuals with significant time constraints	Improved Adherence: Asynchronous or flexible home-based models significantly increase uptake rates compared to rigid, facility-based schedules.
Clinically Stable	Exclusion of subjects with unstable angina or severe arrhythmias	Safety and Latency: In the absence of direct physical supervision, the primary clinical concern remains the potential latency in emergency response for high-risk patients.
No CIEDs (Pacemaker/ICD)	Patients without implanted cardiac electronic devices	Evidence Gap: The safety of wearable sensors regarding electromagnetic interference is not yet fully validated in this specific sub-population.
Intact Cognitive Status	Patients without cognitive impairment or severe sensory deficits	Self-Management Requirement: CTR efficacy relies on the patient’s ability to self-monitor and accurately respond to digital feedback and alerts.

CIEDs: cardiac implantable devices; CR: cardiac rehabilitation; CTR: cardiac telerehabilitation; CV: cardiovascular; FDA: Food and Drug Administration; HFrEF: heart failure with reduced ejection fraction; PCI: percutaneous coronary interventions.

## Data Availability

No new data were created or analyzed in this study. Data sharing is not applicable to this article.
